# Three-Phase Equilibria of CO_2_ Hydrate from Computer Simulation in the Presence of NaCl

**DOI:** 10.1021/acs.energyfuels.5c00174

**Published:** 2025-03-10

**Authors:** A. Borrero, A. Díaz-Acosta, S. Blazquez, I. M. Zerón, J. Algaba, M. M. Conde, F. J. Blas

**Affiliations:** † Laboratorio de Simulación Molecular y Química Computacional, CIQSO – Centro de Investigación en Química Sostenible and Departamento de Ciencias Integradas, 16743 Universidad de Huelva, 21006 Huelva, Spain; ‡ Departamento de Química Física, Facultad de Ciencias Químicas, 16734 Universidad Complutense de Madrid, 28040 Madrid, Spain; § Departamento de Ingeniería Química Industrial y Medio Ambiente, Escuela Técnica Superior de Ingenieros Industriales, Universidad Politécnica de Madrid, 28006 Madrid, Spain

## Abstract

In this work, the cryoscopic decrease effect, as a function of the NaCl concentration, on the carbon dioxide (CO_2_) hydrate dissociation line conditions was determined through molecular dynamic simulations. In particular, we have determined the three-phase (solid hydrate–aqueous phase–liquid CO_2_) coexistence temperature at 100, 400, and 1000 bar at several initial NaCl concentrations in the aqueous phase, from 0.0 to 3.0 m, using the direct-coexistence technique. We used the well-known TIP4P/2005 and TraPPe force fields for water and CO_2_ molecules, respectively. Also, the water–salt interactions were described using the Madrid-2019 force field, which has been specifically developed for various salts in combination with the TIP4P/2005 water model. According to the results obtained in this work, the dissociation temperature of the CO_2_ hydrate decreases when the NaCl concentration in the initial aqueous phase increases. The results obtained are in excellent agreement with the experimental data reported in the literature. We have also observed how the dynamics of melting and growth of the CO_2_ hydrate becomes slower when the NaCl concentration is increased. As a consequence, longer simulation times (on the order of dozens of microseconds) are necessary when the NaCl concentration increases. Finally, we have also analyzed finite-size effects on the three-phase coexistence temperature of these systems by performing simulations at 400 bar with two different system sizes at two different NaCl concentrations (0.0 and 3.0 m). Non-negligible deviations have been found between the results obtained from the two system sizes.

## Graphical abstract

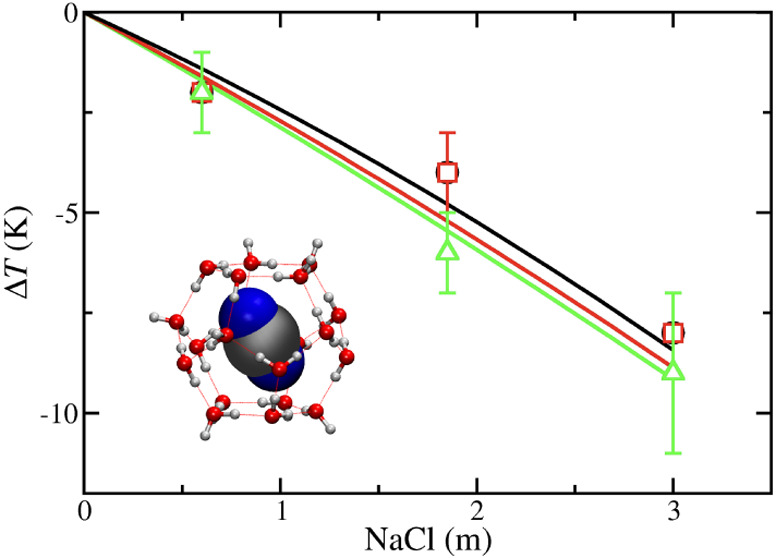

## Introduction

Clathrates are nonstoichiometric crystalline inclusion compounds in which small guest molecules, such as methane (CH_4_), carbon dioxide (CO_2_), hydrogen (H_2_), or nitrogen (N_2_), and medium guest molecules, such as ethane (C_2_H_6_), propane (C_3_H_8_), iso-butane (C_4_H_10_), or tetrahydrofuran (THF), among numerous other species, are enclathrated within the voids left by a periodic arrange of hydrogen-bonded particles (host).^1,2^ When the network of hydrogen-bonded molecules is built with water molecules (H_2_O), clathrates are called clathrate hydrates or simply hydrates.^1,2^ In the last decades, hydrates have been the subject of fundamental and applied research^1–4^ because of their promising applications in CO_2_ capture,^5–14^ H_2_ storage and transport,^15–17^ N_2_ recovery from industrial emissions,^18,19^ H_2_S as hydrate promoter,^20^ and also from an energetic point of view since there is more CH_4_ trapped in hydrates in the nature than in conventional fossil fuel reservoirs.^21,22^ Recent estimates predict the existence of 3000 trillion cubic meters of natural gas in the form of hydrates. This is much larger than the 600–850 trillion cubic meters available from conventional sources.^23^ A large amount of CH_4_ hydrate reservoirs have been known for decades in different parts of the world.^24,25^ However, only in recent years, countries such as the USA and Japan have initiated pilot plants to extract CH_4_ from seabed hydrates.^1^ Obviously, the efficient and optimal exploitation of CH_4_ from these resources requires, among many other disciplines and technology development, a precise knowledge of its thermodynamics, including phase equilibrium as well as interfacial and dynamic properties.

As has been mentioned previously, from an environmental point of view, hydrates are also valuable and strategic materials in the field of CO_2_ capture^5,6,8–13^ and storage.^14^ The increase in the amount of CO_2_ in the Earth’s atmosphere, mainly due to human industrial activity, is one of the main sources of climate change and radically affects our society on a planetary scale. The possibility of using sI-hydrates for CO_2_ storage has been considered for decades due to their thermodynamic stability under relatively mild conditions.^1,2^ In this context, it is of great interest the possibility of recovering CH_4_ from ocean floor CH_4_ hydrates at the same time that CO_2_ is stored in situ.^26,27^ This is possible since CO_2_ hydrates are more stable than CH_4_ hydrates at natural gas reservoirs’ pressure and temperature conditions on the seafloor. However, in order to collect CH_4_ from seafloor hydrates at the same time that CO_2_ is stored in situ, it is necessary to obtain a precise knowledge of the thermodynamic, phase equilibrium, interfacial, and dynamic properties of CH_4_ and CO_2_ hydrates, not only at the temperature and pressure conditions of the seafloor but also at the salinity conditions of seawater. For this reason, it is essential to locate the so-called dissociation line or three-phase coexistence line, hydrate-aqueous phase, CO_2_/CH_4_, in a pressure–temperature phase diagram at the salinity conditions of seawater. Precise knowledge of the three-phase coexistence line provides information about the pressure and temperature conditions at which the hydrate is stable. The use of molecular dynamics simulations to study the effect of the NaCl concentration on the CH_4_ hydrate dissociation conditions has already been reported in the literature. Yagasaki et al.^28^ performed molecular dynamic simulations at *m* = 0.6 and 4.8 kg/mol to study the dissociation kinetics of the CH_4_ hydrate at three different temperatures over the dissociation one. They find that NaCl could have both deceleration and acceleration effects on the kinetics of hydrate dissociation. In particular, they observed an acceleration effect at the *m* = 4.8 kg/mol NaCl concentration, which corresponds to the saturated conditions at ambient temperature and pressure, and temperatures close to the CH_4_ hydrate dissociation one. Very recently, Blazquez et al.^29^ have determined the three-phase coexistence temperature for a methane hydrate system in equilibrium with a NaCl solution and a methane gas phase. The study has been performed at 400 bar and a range of concentrations given in molality, below 4 m. In this work, we concentrate on the determination of the three-phase CO_2_ hydrate-aqueous solution-CO_2_-rich liquid phase or dissociation line with different modalities up to 3 m.

Seawater is a complex aqueous solution composed of many components, including a large number of ions. However, most of the physical properties of seawater are essentially dependent on a single parameter: salinity, i.e., the concentration of ions in water. Indeed, salinity is one of the fundamental properties of seawater and is key to understanding biological and physical processes in the oceans. Absolute salinity is defined as the ratio of the mass of dissolved material in seawater to the mass of seawater^30^ (in grams per kilogram of seawater). The salinity of the oceans is typically between 31 and 38 g/kg. According to the International Association for the Physical Sciences of the Oceans (IAPSO),^31,32^ the reference composition of standard seawater is defined in terms of the molar fractions of its 15 major constituents, including water, with the main constituent being sodium chloride (NaCl).^32,33^ In particular, sodium and chloride ions account for up to 90% of the total electrolytes dissolved in water. Therefore, in most studies, seawater is modeled as an aqueous solution of NaCl with a salinity of about 35 g/kg. This concentration is often expressed in terms of molality, i.e., the number of moles of NaCl per kilogram of solvent (water). In the case of seawater, a NaCl salinity of 35 g/kg is equivalent to a NaCl molality of 0.6 m.

From an experimental point of view, there are several studies in the literature that have determined the dissociation line of the hydrate of CO_2_ not only in the presence of seawater but also at higher-salinity conditions, reaching molalities up to 5 m.^34–38^ In particular, experimental studies show that the presence of NaCl in water causes the well-known cryoscopic lowering or melting point depression of the CO_2_ hydrate. In particular, the dissociation temperature of this hydrate, when it is in contact with a NaCl 0.6 m aqueous solution, is approximately 2 K lower than when the NaCl molality of the aqueous solution is zero.^34,38^ The dissociation temperature decreases when the amount of NaCl in the aqueous phase increases. Unfortunately, molecular simulation studies to determine the effect of seawater salinity on the dissociation line of the CO_2_ hydrate are scarce. One of the few found is the recent work of Vlugt and co-workers about the microscopic behavior of CO_2_ hydrates in oceanic sediments.^39^ At this point, an interesting question arises: are the molecular models available in the literature able to reproduce the cryoscopic decrease of the CO_2_ hydrate dissociation line due to the presence of dissolved salts in water through molecular simulations? In a very recent work, Blazquez et al.^40^ have determined the solubility of CO_2_ in salty water through molecular dynamic simulations. In particular, they found that the combination of the TIP4P/2005,^41^ Madrid-2019,^42,43^ and TraPPE-UA^44^ models for water, NaCl-water interactions, and CO_2_, reproduces accurately the density of the aqueous phase as well as the solubility and the salting out effect of the CO_2_ in salty water. This combination of molecular models used in the work of Blazquez et al.^40^ is far from being arbitrary. The TIP4P/2005 ^41^ water and TraPPE-UA^44^ CO_2_ models have been used previously by Míguez et al.^45^ to determine the dissociation temperature of the CO_2_ hydrate. Although in the work of Míguez et al.^45^ they conclude that the results predicted by the mentioned model combination were about ≈30–45 K lower than the experimental ones, they were able to reproduce qualitatively the dissociation line of the CO_2_ hydrate. In the same work, they conclude that the TIP4P/Ice^46^ water model provides better predictions for the dissociation condition of the CO_2_ hydrate. Unfortunately, the Madrid-2019 ^42,43^ NaCl model has been specifically designed for the TIP4P/2005 ^41^ water model. For this reason, in this work, we have decided to use TIP4P/2005 instead of the TIP4P/Ice water model since we are more interested in accurately analyzing the cryoscopic lowering effect on the CO_2_ hydrate than in quantitatively determining the CO_2_ hydrate dissociation temperature in salty water.

The organization of this paper is as follows: In the next section, we describe the simulation details and the methodology. The results obtained in this work at the different thermodynamic conditions and NaCl concentrations are discussed in detail in the Results section. Finally, conclusions are presented in the last section.

## Methodology

### Simulation Details and Molecular Models

In this work, all molecular dynamics simulations have been carried out using GROMACS (version 4.6.5, double precision).^47^ Our system is composed of three components: water, CO_2_, and NaCl. It is essential to describe the interactions accurately. Water molecules have been modeled using the well-known rigid and nonpolarizable TIP4P/2005 ^41^ model. For the CO_2_ molecules, we employed the TraPPE^44^ (Transferable Potentials for Phase Equilibria) force field. This model has been successfully tested for the phase equilibria of CO_2_ hydrates.^48–50^ To describe the water–salt interactions in our system, we have used the Madrid-2019 force field.^42,43^ This force field has been developed for various salts in combination with TIP4P/2005, assigning partial charges to the ions (e.g., ± 0.85e for Na^+^ and Cl^–^).

It is worth noting that the combination of the TIP4P/2005 water model and the TraPPE CO_2_ model has previously been used to study the three-phase coexistence line of CO_2_ hydrate in the absence of salt.^45^ These results showed significant deviations from the experimental data available in the literature. In their work, Míguez et al.^45^ extended their study by also using the TIP4P/Ice^46^ model, which resulted in a remarkable improvement in their results. This improvement is not coincidental: Conde and Vega^51^ previously demonstrated that, for methane hydrates, there is a correlation between the melting temperature of ice *I*_h_ (*T*_m_) and the three-phase coexistence temperature (*T*_3_) of the methane hydrate. Models that accurately predict the melting point of ice *I*_h_, such as TIP4P/Ice, are better suited to reproducing the experimental hydrate equilibrium line.

However, the goal of the present work is to determine the three-phase equilibrium of the CO_2_ hydrate in the presence of salt. Recent studies have demonstrated that using a model with partial charges improves the prediction of the equilibrium line at both intermediate and high salt concentrations, as shown for systems involving ice *I*_h_,^52,53^ methane hydrate,^29^ and even the role of static electric fields in seawater icing.^54^ These findings support our decision to use the Madrid-2019 force field with partial charges, which, in combination with the TIP4P/2005 model, has demonstrated significant superiority in describing a wide range of properties of aqueous solutions compared to unit charge models.^40,43,55–58^ In fact, when using other force fields for the study of the cryoscopic decrease of the *T*_3_ of methane hydrate such as Joung–Cheatham^59^ or Smith and Dang^60^ models, the effect of the salt force field has been negligible at low concentrations.^29,61^

When salt is added to the aqueous phase, the thermodynamic conditions for dissociation of the CO_2_ hydrate are modified. The three-phase coexistence temperature shifts depending on the amount of NaCl added to the aqueous solution phase. Specifically, we are interested in the deviation of *T*_3_ as a function of the molality of NaCl in the aqueous solution, rather than the absolute value of *T*_3_. The aim of this work is to study the cryoscopic effect on freezing-point depression as a function of NaCl concentration. For this reason, we employed the Madrid-2019 force field, where the nonbonded interaction parameters of NaCl have been optimized for the TIP4P/2005 water model. A summary of the nonbonded interaction parameters of the molecular models is presented in Table 1. The Lennard-Jones (LJ) parameters for interactions not listed in Table 1 are determined by using the Lorentz–Berthelot rules. Note that the Lorentz–Berthelot rule is not applied to water–ion or ion–ion interactions in the Madrid-2019 force field.

**Table 1:** Nonbonded Interaction Parameters of Water, CO_2_, and NaCl Models Used in This Work^a^

atom	*q* (e)	σ (Å)	ε (kJ/mol)
Water			
O_w_		3.1589	0.7749
H	+0.5564		
M	–1.1128		
CO_2_			
C	+0.70	2.80	0.224478
O	–0.35	3.05	0.656806
NaCl			
Na^+^	+0.85	2.21737	1.472356
Cl^–^	–0.85	4.69906	0.076923
Na^+^–Cl^–^		3.00512	1.438894
Na^+^–O_w_		2.60838	0.793388
Cl^–^–O_w_		4.23867	0.061983

a Water molecules are described using the TIP4P/2005 model.^41^ For NaCl, we use the parameters from Madrid-2019 force field,^42,43^ and LJ interaction parameters for CO_2_ are taken from ref 44.

All molecular dynamic simulations have been carried out in the *NPT* ensemble. In order to avoid any stress from the solid CO_2_ hydrate structure, volume fluctuations are performed independently in each space direction. The three-phase coexistence temperature, *T*_3_, was estimated at three different pressures (100, 400, and 1000 bar) and several NaCl concentrations (0, 0.6, 1.85, and 3.0 m). The *T*_3_ value at each pressure and concentration has been obtained by using the direct-coexistence technique.^45,62–64^ Following this approach, the hydrate phase, the aqueous solution phase (with different NaCl concentrations), and the liquid CO_2_ phase are put together in the same simulation box. By keeping constant the pressure, *P*, and performing simulations at different temperatures, *T*, it is possible to evaluate the temperature at which the three phases coexist in equilibrium, *T*_3_. If *T* > *T*_3_ then, the initial three-phase system evolves to a two-phase system since the hydrate phase melts releasing water and CO_2_ molecules to the aqueous solution and liquid CO_2_ phases. Contrary, if *T* < *T*_3_, then the hydrate phase will grow. Here it is important to take into account that due to the presence of NaCl in the aqueous solution, the hydrate phase will not grow until extinguishing the aqueous solution phase since the concentration of NaCl in the aqueous solution increases at the same time that the molecules of water move from the aqueous solution to the hydrate phase. As a consequence, the system evolves from the initial configuration to a hydrate phase in equilibrium with the liquid CO_2_ phase and, also, in equilibrium with a supersaturated NaCl aqueous solution phase. We consider that *T* < *T*_3_ if a couple of extra slabs of hydrates are formed even if we do not observe the complete crystallization of the aqueous solution phase. The *T*_3_ value at a given initial NaCl concentration is obtained as the middle temperature between the highest temperature at which the hydrate phase grows and the lowest temperature at which the hydrate phase melts.

In order to maintain constant temperature and pressure, the v-rescale thermostat^65^ and the anisotropic Parrinello–Rahman barostat^66^ are used, both with a time constant of 2 ps. For the Parrinello–Rahman barostat, a compressibility value of 4.5 × 10^–5^ bar^–1^ is applied anisotropically in all three directions of the simulation box. Dispersive and Coulombic interactions are truncated by using a cutoff value of 1.0 nm in both cases. No long-range corrections or modifications to the Lorentz–Berthelot combining rule are applied for the dispersive interactions between water and CO_2_ molecules. For the Coulombic interactions, long-range particle mesh Ewald (PME) corrections^67^ are employed with a mesh width of 0.1 nm and a relative tolerance of 10^–5^.

### Box Size

In this work, we used two types of simulation box sizes: a smaller box labeled as S and a larger box labeled as L. Specifically, we chose the S-size configuration to compare with the results for *T*_3_ from the original work without salt performed by Míguez et al.^45^ They studied the three-phase coexistence line of the CO_2_ hydrate at different pressures using the same potential parameters for water and CO_2_ as in the present work. The initial simulation box used in the work of Míguez et al.^45^ was built by replicating the CO_2_ hydrate unit cell twice in each space direction (2 × 2 × 2). The total number of molecules of water and CO_2_ in the hydrate phase was 368 and 64, respectively. Then, the same number of molecules of water was used in the aqueous phase, and finally, 192 molecules of CO_2_ were placed in the liquid CO_2_ phase. We followed the same protocol to build the hydrate phase of the initial S configuration in this work. However, we increased the number of molecules in the aqueous phase and the liquid CO_2_ phase to 1110 and 320 water and CO_2_ molecules, respectively. This adjustment was necessary because we now include NaCl molecules in the aqueous phase. If the aqueous phase is too small and *T* < *T*_3_, when the hydrate phase grows and the molecules of water move from the aqueous solution to the hydrate phase, the concentration of NaCl increases rapidly avoiding the expansion of the hydrate phase. Thus, we have increased the number of water molecules in the initial aqueous solution by a ∼3 factor in the S configuration compared to the original work of Míguez et al.^45^

As demonstrated recently by some of us,^48–50^ the *T*_3_ value determined using the direct-coexistence technique is not free of finite-size effects. Therefore, it is crucial to use systems that are large enough to accurately determine the impact of the NaCl concentration on the *T*_3_ value. In order to avoid finite-size effects, we built a large initial simulation box (L) by replicating the CO_2_ hydrate unit cell three times in each space direction (3 × 3 × 3). The numbers of water and CO_2_ molecules in the initial hydrate phase are 1242 and 216, respectively. We have also increased the number of water and CO_2_ molecules in both the aqueous solution and liquid CO_2_ phases. In the case of the aqueous solution phase, we doubled the number of water molecules relative to the hydrate phase (2484 water molecules in the aqueous solution). Additionally, we placed 1000 CO_2_ molecules in the liquid CO_2_ phase. As it has been demonstrated recently,^48,49^ this arrangement regarding the number of molecules in the simulation box prevents the formation of a liquid CO_2_ drop within the aqueous solution. The formation of a liquid drop or a gas bubble leads to an incorrect determination of *T*_3_, allowing growth of the hydrate phase at temperatures above the real *T*_3_ value. A summary of the number of molecules in each phase for the two configurations (S and L) and the initial NaCl concentrations in the aqueous phase for each configuration can be found in Table 2.

**Table 2:** Initial Number of Water, CO_2_, and NaCl Molecules in the Three Phases Involved in the Equilibrium for the L and S Configurations at Each NaCl Concentration

		hydrate phase			aqueous phase			liquid CO_2_ phase
configuration	NaCl concentration (m)	unit cell	water	CO_2_	water	Na^+^	Cl^–^	CO_2_
L	0	3 × 3 × 3	1242	216	2484	0	0	1000
	0.6					27	27	
	1.85					83	83	
	3.0					134	134	
S	0	2 × 2 × 2	368	64	1110	0	0	320
	3.0					60	60	

## Results

In this section, we present the different *T*_3_ results obtained as a function of the NaCl concentration in the aqueous phase and the pressure. We also analyze the effect of the simulation box size on the *T*_3_ values at 400 bar when the initial NaCl concentrations are 0 and 3.0 m. Also, we analyze the effect of the NaCl concentration on the dynamics of growth and melting of the CO_2_ hydrate. Finally, we compare the cryoscopic decrease effect, as a function of the NaCl concentration, on the CO_2_ hydrate obtained from the simulation with experimental data taken from the literature.

### NaCl 0.0 m

First of all, we determine the three-phase coexistence temperature, *T*_3_, at 100, 400, and 1000 bar without the presence of NaCl in the aqueous solution phase using configuration L, and at 400 bar using configuration S. Since we are going to determine the change in the *T*_3_ value as a function of the NaCl concentration, we need to know the exact *T*_3_ value without the presence of NaCl. As it has been mentioned previously, in the work of Míguez et al.,^45^ they determined the *T*_3_ of the CO_2_ hydrate using, as well as in this work, the TIP4P/2005 ^41^ and TraPPE^44^ models to describe the molecules of water and CO_2_ respectively. However, the total number of molecules used by Míguez et al.^45^ is different than that used in this work. As has been demonstrated by some of us in previous work,^49^ finite-size effects on the *T*_3_ determination of the CO_2_ hydrate become negligible when nonstoichiometric configurations with larger unit cells, like 3 × 3 × 3 and 4 × 4 × 4, are used. It means that the *T*_3_ values obtained by using configurations L and S can be different from that obtained in the original work of Míguez et al.^45^ Since we are interested in determining how the *T*_3_ of the CO_2_ hydrate is affected by the NaCl concentration in the aqueous phase, first, it is necessary to determine accurately the *T*_3_ value with both simulation boxes used in this work. For this reason, the *T*_3_ values at 100, 400, and 1000 bar have been calculated using configuration L and, also, the *T*_3_ at 400 bar has been calculated using configuration S. In both cases, the initial aqueous solution phase was formed only by molecules of water.

The potential energy as a function of the simulation time obtained from configuration L at 100, 400, and 1000 bar is represented in Figure 1. The *T*_3_ values obtained using configuration L at 100 (a), 400 (b), and 1000 (c) bar are 247(1), 249(1), and 253(1) K, respectively. As can be observed, the *T*_3_ values obtained in this work by using configuration L and the results obtained by Míguez et al.^45^ are the same within the error bars (249(2), 249(2), and 252(2) K at 100, 400, and 1000 bar, respectively). As we have pointed out previously, the size and the number of molecules in configuration L is much higher than that used in the work of Míguez et al.,^45^ so one could expect different results. However, it is important to note that the cutoffs employed in both works are different. In the work of Míguez et al.,^45^ simulations were carried out using a cutoff value of 0.9 nm and homogeneous long-range corrections for the Lennard-Jones (LJ) dispersive interactions, while in this work the value of the cutoff is 1.0 nm and we have not employed long-range corrections for the LJ interactions. Some of us have demonstrated in a very recent work^50^ the effect of the cutoff and long-range corrections on the hydrate *T*_3_ determination. The use of homogeneous long-range corrections increases the *T*_3_ value of the system. However, one should be careful when they are applied to a system where different phases coexist since, technically, they can be only applied to homogeneous systems. In this particular case, the use of homogeneous long-range corrections compensates for the small size of the system used in the original work of Míguez et al.^45^ This explains why the results obtained in this work using configuration L are similar to those obtained by Míguez et al.^45^ even when the size of the simulation boxes are different. A summary of the results obtained in this work is presented in Table 3.

**Figure 1:**
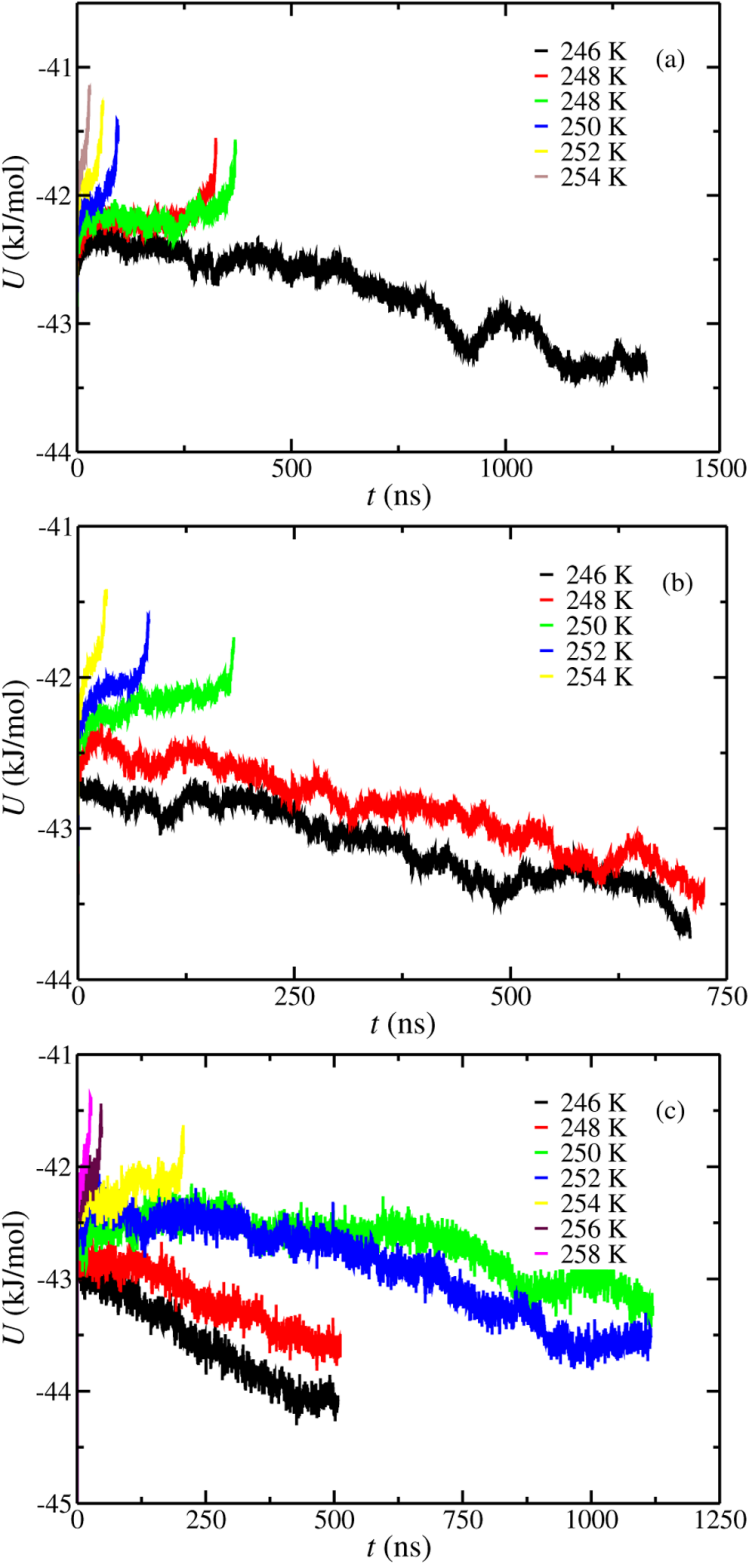
Potential energy as a function of time as obtained from molecular dynamic simulations at different temperatures and pressures using configuration L simulation box. (a–c) Results obtained at 100, 400, and 1000 bar, respectively. In all cases, the initial aqueous phase is a pure water phase without NaCl. The temperatures studied at each pressure are represented in the legend.

**Table 3:** *T*_3_ (K) Values as a Function of Pressure and the NaCl Concentration (m) as Obtained from Molecular Dynamics Simulations Using Configuration L

		temperature (K)		
pressure (bar)	0.0 m	0.6 m	1.85 m	3.0 m
100	247(1)	245(1)	243(1)	239(1)
400	249(1)	247(1)	245(1)	241(1)
1000	253(1)	251(1)	247(1)	244(2)

Also, we determine the *T*_3_ value at 400 bar using configuration S as shown in Figure 2. The *T*_3_ predicted using configuration S is 245(2) K. As observed, there are differences between the *T*_3_ values obtained using configurations L and S at 400 bar. It is important to recall that simulations have been carried out at the same conditions and using the same simulation details except for the simulation box size. It means that the difference in the *T*_3_ value is caused by finite-size effects and the inherited stochasticity of the direct-coexistence methodology. Also, it is interesting to compare this result with the results obtained in the work of Míguez et al.^45^ at 400 bar, 249(2) K. The system size used in configuration S is closer to that used in the original work of Míguez et al.^45^ than the system size used in configuration L. However, the result obtained by using configuration S is different from that reported in the work of Míguez et al.^45^ As it has been pointed out previously, the cutoff employed in both works is different, and this explains why the results obtained in both works are different even when the system sizes are similar.

**Figure 2:**
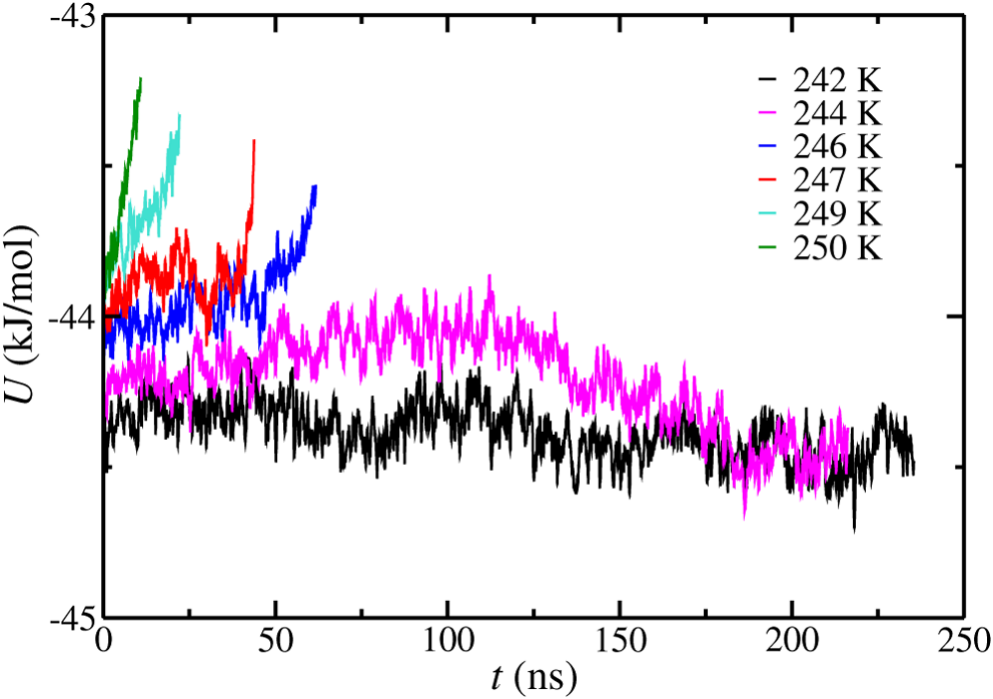
Potential energy as a function of time as obtained from molecular dynamic simulations at different temperatures at 400 bar using configuration S simulation box. In all cases, the initial aqueous phase is a pure water phase without NaCl.

### NaCl 0.6 m

Now, we study the effect of a 0.6 m NaCl concentration in the aqueous phase on the *T*_3_ values obtained at 100, 400, and 1000 bar using configuration L. The 0.6 m NaCl concentration is especially interesting, since it corresponds to the natural NaCl concentration of the seas and ocean. Most hydrate studies are carried out without taking into account the presence of NaCl in the aqueous phase. However, hydrates are common on the seabed, and for this reason, is essential to understand how the stability conditions of these compounds are modified by the presence of NaCl. The potential energy as a function of the simulation time obtained from configuration L at 100, 400, and 1000 bar is represented in Figure 3. The *T*_3_ values obtained using configuration L at 100 (a), 400 (b), and 1000 (c) bar are 245(1), 247(1), and 251(1) K, respectively. A summary of the *T*_3_ results is presented in Table 3. As observed in Figure 3 and Table 3, the *T*_3_ value is systematically reduced by 2 K at each pressure considered in this work when the NaCl concentration is increased from 0.0 to 0.6 m. This is the expected behavior since the presence of NaCl in the aqueous phase decreases the *T*_3_ of the hydrates.

**Figure 3:**
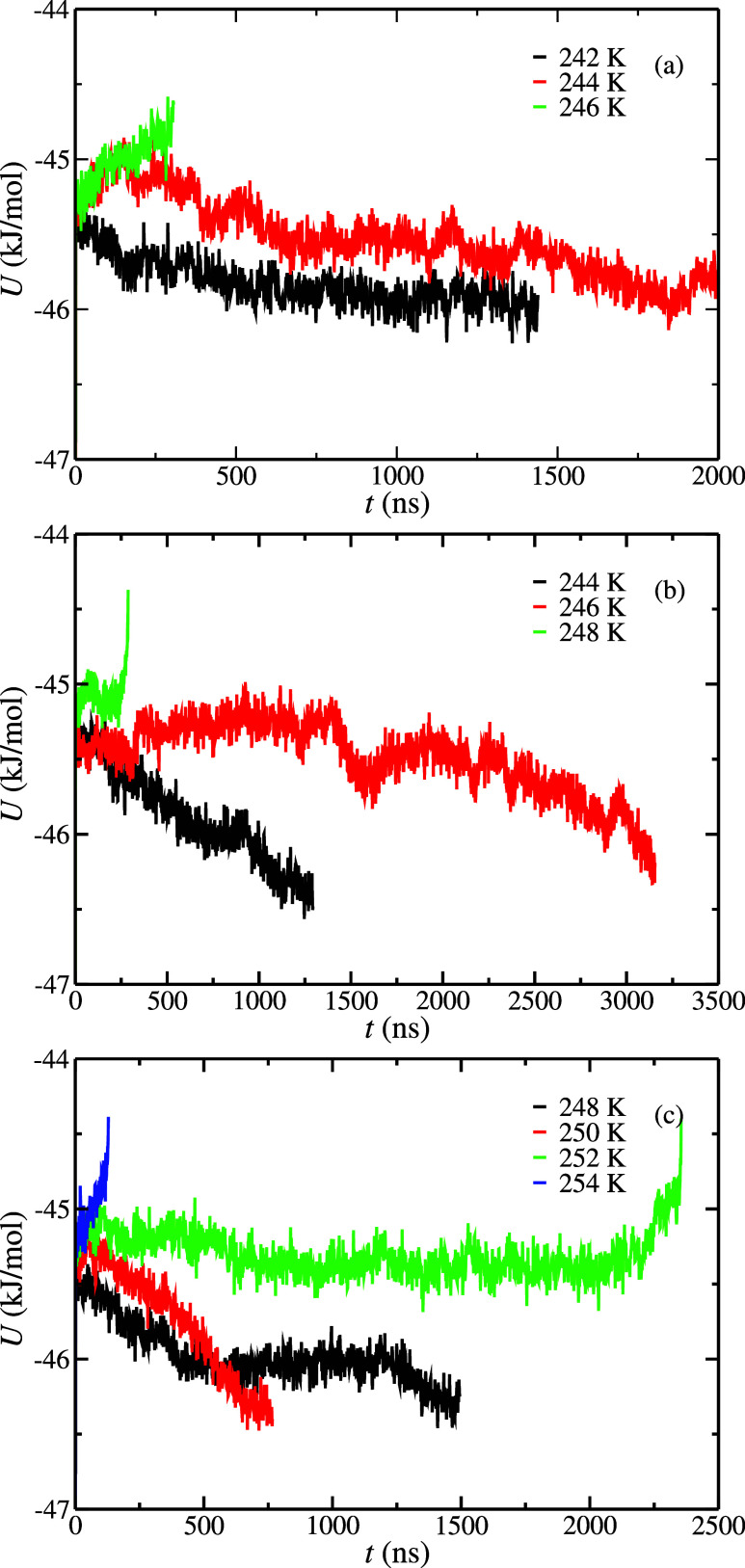
Potential energy as a function of time as obtained from molecular dynamic simulations at different temperatures and pressures using configuration L simulation box. (–c) correspond to results obtained at 100, 400, and 1000 bar, respectively. In all cases, the initial aqueous phase had a 0.6 m NaCl concentration. The different temperatures are represented in the legend.

It is also interesting to analyze the behavior of the dynamics of the system as a function of NaCl concentration. As can be observed in Figures 1 and 3, simulation times become larger when the NaCl concentration is increased. Yagasaki et al.^28^ reported a similar slowing down effect at a NaCl 0.6 m concentration for the CH_4_ hydrate dissociation case. In general, we can conclude that simulation times when the NaCl concentration is 0.6 m are about 1.5–2.0 times larger than those required in the absence of NaCl. It is also interesting to remark that larger hydrate systems required, in general, larger simulation times.^48,49^ This is important since an increment in the size of the system yields larger simulation times. Also, simulations become more expensive when the size of the system is increased. Finally, as has been pointed out previously, the presence of NaCl in the aqueous phase affects the dynamics of the hydrates, and larger simulation times are required. This phenomenon is due to the fact that the salt acts as an inhibitor of the formation of the hydrate. The Na^+^ cations and Cl^–^ anions solvate the molecules of water, forming a hydration sphere around the ions, resulting in a reduction of the water molecules available to form the CO_2_ hydrate structure under crystallization conditions (*T* < *T*_3_). Another reason is that the solubility of CO_2_ in the aqueous phase decreases when the salinity is increased.^40,68^ In the work of Sun et al.,^68^ they calculated the solubility of CO_2_ in water and in saline solutions in equilibrium with CO_2_ hydrates. In this study, it is observed that the solubility of CO_2_ in water decreases as the concentration of NaCl in the aqueous phase increases. This has a double effect, on the one hand, under dissociation conditions, the CO_2_ releases from the dissociation of the hydrate can not pass through the aqueous solution to reach the CO_2_ phase, leading to the formation of the hydrate again and delaying the dissociation of the hydrate. On the other hand, this phenomenon also increases simulation times because the low solubility of CO_2_ in saline solutions makes it difficult for the CO_2_ molecules to pass from the CO_2_ phase to the hydrate surface (through the aqueous phase), delaying the formation of new layers of CO_2_ hydrate. In summary, one could be tempted to use smaller and more affordable system sizes to study how the presence of NaCl affects the dissociation temperature of the hydrates. However, small systems are affected by finite-size effects and can not be used to determine accurately the dissociation temperature of these systems.^48,49^

### NaCl 1.85 m

In this section, we determine the three-phase dissociation temperature, *T*_3_, of the CO_2_ hydrate when the initial aqueous phase has a concentration of 1.85 m of NaCl. We have analyzed this system using configuration L. As we have shown previously, the amount of NaCl in the aqueous solution has an effect on the *T*_3_ as well as the simulation time required by the system to evolve. As can be observed in Figure 4 and in Table 3, the dissociation temperature as a function of the NaCl concentration behaves as expected; when the NaCl concentration is increased, the *T*_3_ value is decreased. In particular, *T*_3_ values at a NaCl concentration of 1.85 m are 243(1), 245(1), and 247(1) K at 100, 400, and 1000 bar, respectively. At 100 and 400 bar, the *T*_3_ decreases 2 K when the NaCl concentration is increased from 0.0 to 0.6 m, and when it is increased from 0.6 to 1.85 m. At 1000 bar, *T*_3_ also decreases when the NaCl concentration is increased. As at 100 and 400 bar, the *T*_3_ value at 1000 bar decreases by 2 K when the NaCl concentration is increased from 0.0 to 0.6 m. However, at 1000 bar, when the NaCl concentration is increased from 0.6 to 1.85 m, the *T*_3_ value decreases 4 K, from 251(1) K at 0.6 m to 247(1) K at 1.85 m.

**Figure 4:**
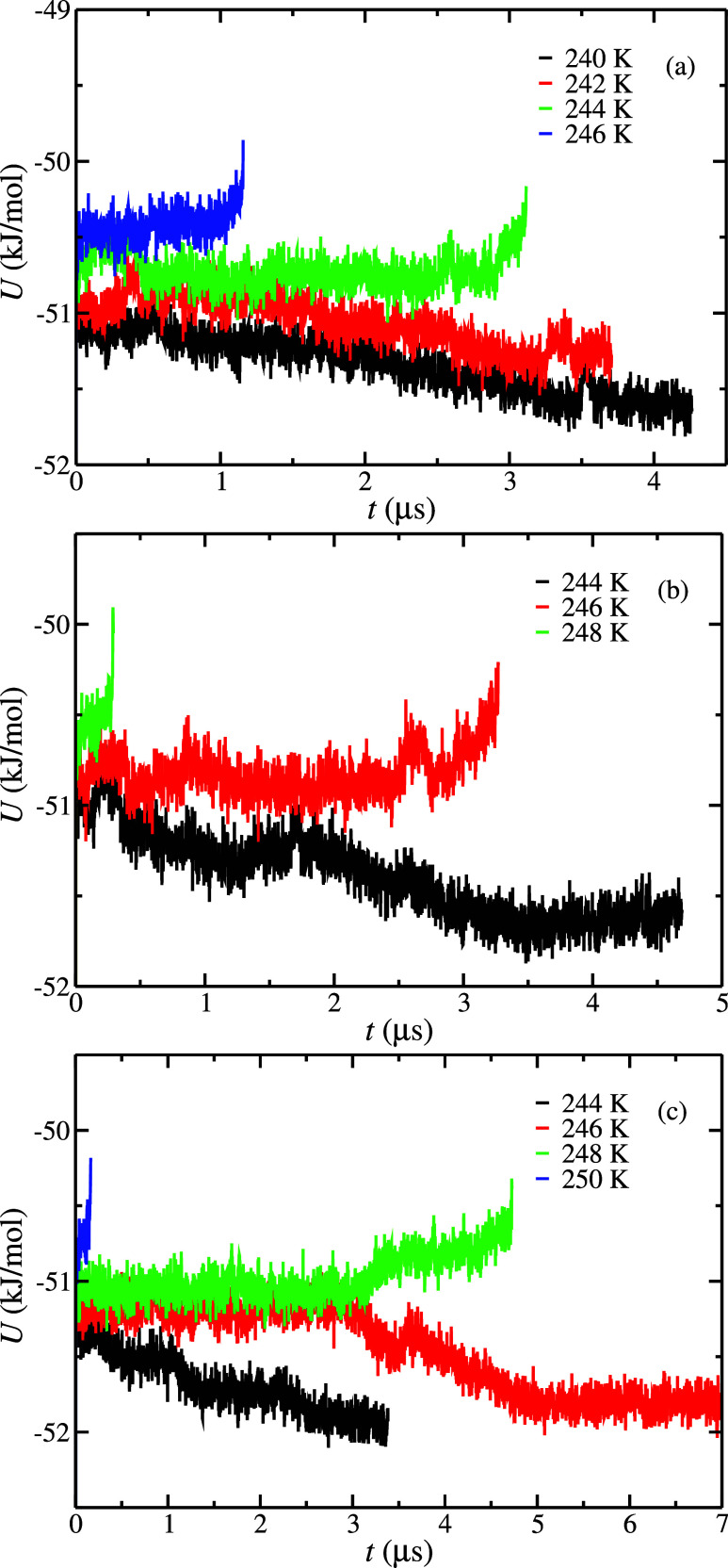
Potential energy as a function of time as obtained from molecular dynamic simulations at different temperatures and pressures using the configuration L simulation box. (a–c) Results obtained at 100, 400, and 1000 bar, respectively. In all cases, the initial aqueous phase has a 1.85 m NaCl concentration. The different temperatures are represented in the legend.

It is also interesting to analyze the dynamics of the system at 1.85 m of NaCl. As has been pointed out previously, when the amount of NaCl in the aqueous phase is increased, the time required for the hydrate to grow or melt is also increased. In particular, simulation times, from 0.6 to 1.85 m, have been increased by a 1.5–2.5 factor. It means that simulation times when the initial aqueous phase has 1.85 m NaCl concentration are about 3–5 times larger than when there is no NaCl in the aqueous phase. Notice that when the temperatures are close to the *T*_3_ values, the simulation time required by the system to evolve is even larger. The amount of NaCl in the aqueous phase affects not only the time required by the hydrate to grow but also to melt. The melting process used to be faster than the growth of the hydrate phase. However, the presence of NaCl slows both processes. As a consequence, it is necessary to increase drastically the simulation time in order to study correctly the behavior of these systems.

### NaCl 3.0 m

Finally, we determine the three-phase dissociation temperature, *T*_3_, of the CO_2_ hydrate when the initial aqueous phase has a concentration of 3.0 m of NaCl. We study the cryoscopic decrease effect at this concentration using configuration L at 100, 400, and 1000 bar. Also, we study the effect of the NaCl concentration at 400 bar using configuration S. First, we focus on the results obtained using configuration L. Figure 5 shows the potential energy as a function of the simulation time at 100 (a), 400 (b), and 1000 (c) bar and different temperatures. As expected, the increase in the salinity of the aqueous phase reduces the *T*_3_ values obtained at each pressure. In particular, the *T*_3_ values obtained at 100, 400, and 1000 bar when the initial aqueous phase has a 3.0 m concentration of NaCl are 239(2), 242(2), and 244(2) K, respectively. If we compare these results with those obtained without the presence of NaCl (0.0 m), we find that *T*_3_ values are reduced by 8, 7, and 9 K at 100, 400, and 1000 bar, respectively. This is in good agreement with the cryoscopic decrease effect reported in the literature.^34,38^ A further comparison with experimental data is presented in the next section.

**Figure 5:**
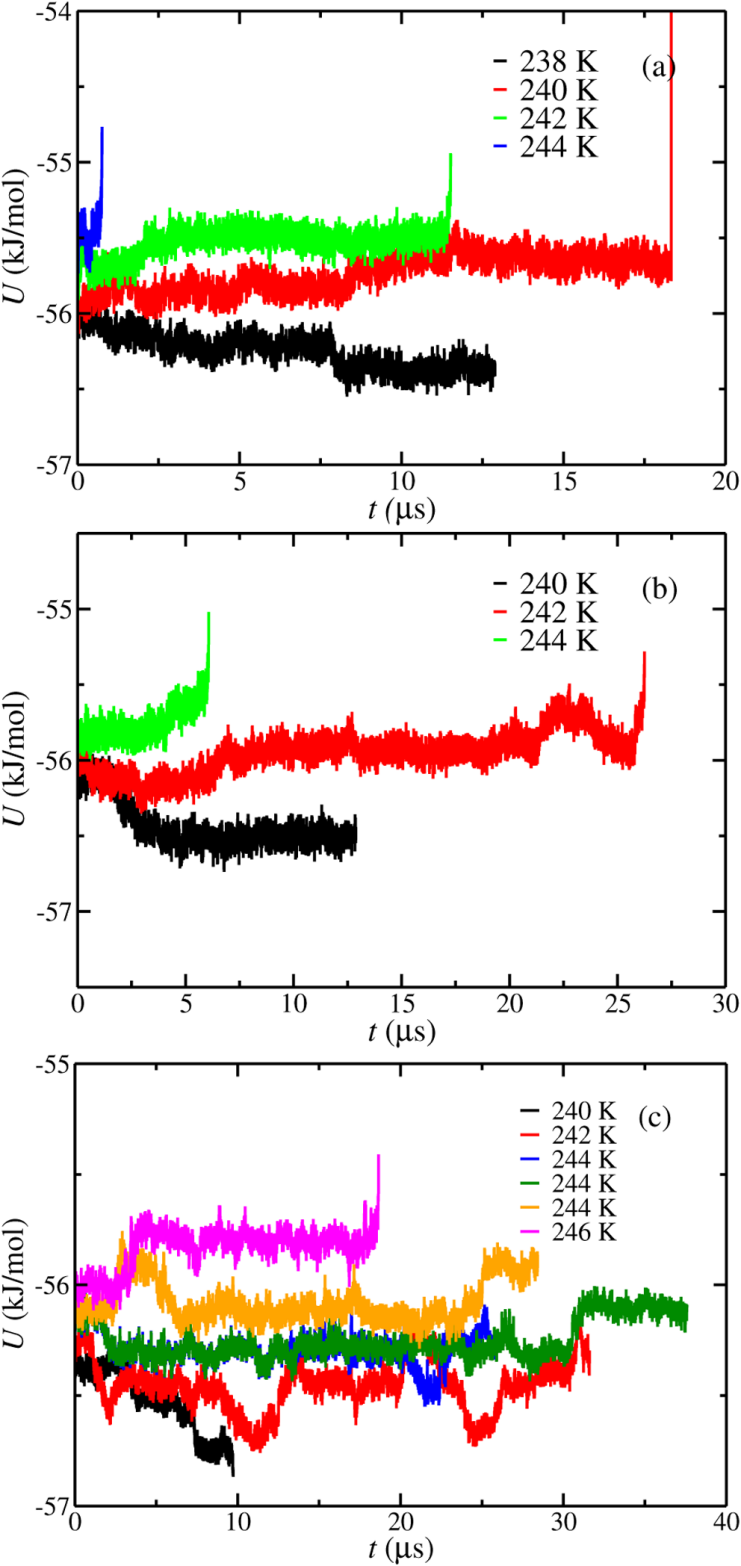
Potential energy as a function of time as obtained from molecular dynamic simulations at different temperatures and pressures using the configuration L simulation box. (a–c) Results obtained at 100, 400, and 1000 bar, respectively. In all cases, the initial aqueous phase has a 3.0 m NaCl concentration. The different temperatures are represented in the legend.

We also studied the cryoscopic decrease effect on the CO_2_ hydrate at 400 bar using configuration S. As can be seen in Figure 6, the *T*_3_ value obtained using configuration S is 240(1) K. Although the values obtained, at NaCl 3.0 m, using configurations L and S are the same within the error bars, the results obtained without the presence of NaCl, at NaCl 0.0 m, are different, 249(1) and 245(1) K, respectively. As a consequence, the predicted cryoscopic decrease effect, i.e., the reduction of the *T*_3_ value as a function of the NaCl concentration, is different using configurations L and S. In particular, the *T*_3_ decreases by 8 and 5 K using configurations L and S, respectively. It means that at 400 bar, the cryoscopic decrease effect obtained from configurations L and S shows a difference of 3 K. Although a 3 K difference could seem negligible, it is important to notice that 3 K represents 60% of the total cryoscopic decrease value obtained from configuration S.

**Figure 6:**
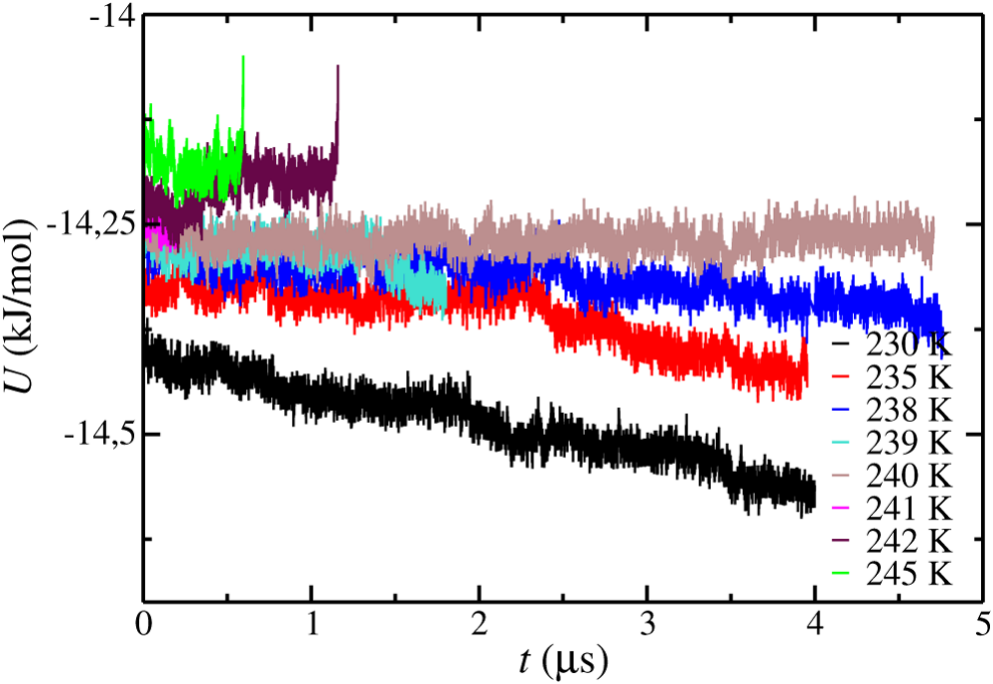
Potential energy as a function of time as obtained from molecular dynamic simulations at different temperatures at 400 bar using configuration S simulation box. In all cases, the initial NaCl concentration in the aqueous phase is 3.0 m.

Finally, we analyze the dynamics of the system with an initial 3.0 m NaCl concentration in the aqueous phase. As shown in Figure 5, the simulation times required to observe the growing/melting behavior of the system become extremely large. In fact, we do not observe any concluding behavior at 1000 bar and *T* = 244 K after running three different seeds for at least 25 μs. It means that simulation times required at 3.0 m become 3–5 times larger than at 1.85 m and, hence, 10–20 times larger than that at 0.0 m. Here it is important to remark that the dynamics of the system becomes slower not only because of the increment of NaCl but also because simulation temperatures become lower due to the cryoscopic decrease effect.

### Cryoscopic Decrease Effect

Finally, we analyze the cryoscopic decrease effect predicted by molecular dynamic simulation. As has been explained previously, the water, TIP4P/2005,^41^ and CO_2_, TraPPE,^44^ molecular models employed in this work are able to reproduce qualitatively the dissociation temperature of the CO_2_ hydrate. The results obtained using these two models underestimate the dissociation temperature by ≈30–45 K. However, in this work, we are interested in the cryoscopic decrease effect, i.e., how the *T*_3_ value is reduced as a function of the salinity. The total temperature reduction, Δ*T*, as a function of the NaCl concentration, can be expressed as the difference between the *T*_3_ value when the initial aqueous solution is pure water minus the *T*_3_ value when the initial aqueous phase contains NaCl at a certain concentration. Notice that when the cryoscopic decrease effect is expressed as Δ*T*, the initial *T*_3_ value becomes irrelevant and allows us to analyze whether the methodology as well as the system sizes and models used in this work are able to reproduce the experimental data.

Figure 7 shows a representation of the cryoscopic decrease effect as a function of the NaCl molality in the aqueous phase. As can be observed, the cryoscopic decrease effect is not a linear function of the molality. The *T*_3_ value shows a poor dependency on the NaCl concentration at low salinity values. However, the slope becomes sharper when the NaCl molality is increased. It is also interesting to analyze the effect of the pressure on the cryoscopic decrease effect. The three pressures studied in this work present the same qualitative behavior. Also, it is interesting to observe that the cryoscopic decrease effect slightly increases when the pressure is increased.

**Figure 7:**
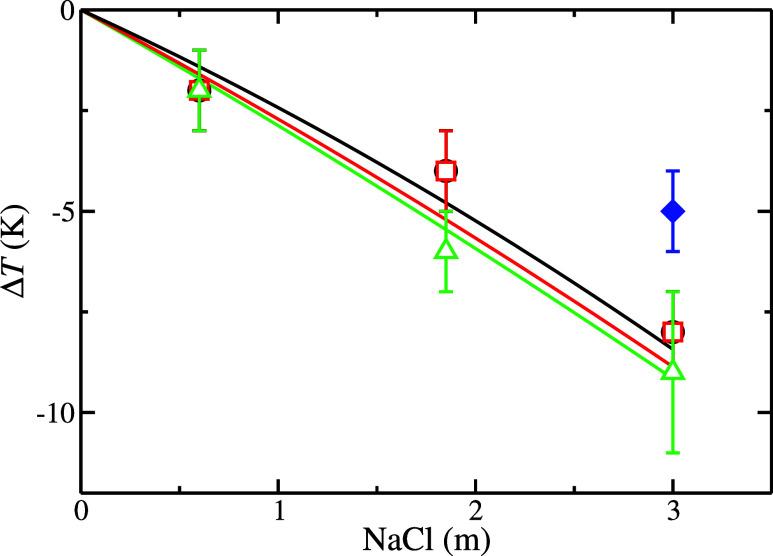
Cryoscopic decrease effect as a function of NaCl molality. Black, red, and green lines correspond to the experimental cryoscopic decrease effect for the CO_2_ hydrate at 100, 400, and 1000 bar, respectively.^38^ Open black circles, red squares, and green up-triangles correspond to the results obtained in this work using configuration L at 100, 400, and 1000 bar, respectively. The filled blue diamond corresponds to the result obtained at 400 bar using configuration S. Notice that black and red symbols (100 and 400 bar) overlap in all cases.

As has been explained previously, the TIP4P/2005 ^41^ and the TraPPE^44^ models for water and CO_2_ have been previously employed to describe the dissociation line of the CO_2_ hydrate.^45^ However, this is the first time that the Madrid-2019 NaCl model^42^ has been employed to describe the dissociation line of a hydrate at different salinity concentrations. As observed, the results obtained in this work using configuration L are in excellent agreement with the experimental results taken from the literature. Also, we can observe that the result obtained by using configuration S underestimates the cryoscopic decrease effect at a NaCl concentration of 3.0 m. Here, it is important to note that the size of the configuration L system is far from being arbitrary. As it has been demonstrated by some of us in a previous series of papers,^48–50^ the size of the simulation box has to be chosen carefully in order to avoid finite-size effects on the *T*_3_ determination. According to our previous works,^48,49^ configuration L is large enough and contains enough molecules to avoid such finite-size effects. However, configuration S should be used carefully since it is too small and the results provided by this configuration are affected by finite-size effects.

## Conclusions

In this work, we used the direct-coexistence simulation technique to study the cryoscopic descent effect on the three-phase dissociation temperature of the CO_2_ hydrate at different initial NaCl molalities in the aqueous phase and several pressures (100, 400, and 1000 bar). Notice that the number of data reported in this work is of the same order of previous studies reported in the literature where the cryoscopic descent effect of ice-like systems is studied through molecular simulation.^29,52,61^ The water and CO_2_ molecules are modeled using TIP4P/2005 and TraPPE force fields, respectively. To describe the water–salt interactions in our system, we have used the Madrid-2019 force field.^42,43^ This force field has been developed for several salts in combination with the TIP4P/2005 water model. According to our previous work,^48,49^ the system L is large enough to avoid finite-size effects. However, we have also performed the same study with a smaller configuration (S) at 400 bar and at NaCl 0 and 3.0 m to analyze finite-size effects on the three-phase dissociation temperature, as well as on the cryoscopic descent effect.

In the absence of NaCl, the *T*_3_ values obtained by configuration L are in good agreement with the data reported previously in the literature.^45^ Contrary, configuration S underestimates the *T*_3_ value at 400 bar by 4 K. Taking into account that the determination of *T*_3_ is the first step in the cryoscopic descent effect study, a reasonable question arises: How much does the size of the system affect the cryoscopic descent? As we demonstrate in this work, the cryoscopic descent effect results at NaCl 0.6, 1.85, and 3.0 m obtained with configuration L are in excellent agreement with the experimental data^38^ reported in the literature in the range of pressure considered in this work. However, the result obtained by using configuration S at 400 bar and 3.0 m NaCl clearly underestimates the cryoscopic descent effect by 3 K which represents 60% of the total cryoscopic decrease value obtained from configuration S.

We also analyze the effect of pressure on the cryoscopic decrease effect using configuration L. As discussed in Figure 7, the cryoscopic decrease obtained in this work from molecular dynamics simulations at 100 and 400 bar is the same at the different NaCl concentrations considered in this study. At 1000 bar, the cryoscopic decrease effect is slightly higher than those obtained at 100 and 400 bar. The simulation results obtained in this work and the previously reported experimental data^38^ are in excellent agreement with the uncertainties. This demonstrated that the combination of the Madrid-2019 force fields^42,43^ (including the TIP4P/2005 water model) and the CO_2_ TraPPE model^44^ provide an accurate description of the cryoscopic decrease effect on the CO_2_ hydrate as a function of the NaCl concentration in the range of pressures considered in this work.

Finally, we also analyze the dynamics of the system, i.e., the simulation times required to observe if the hydrate phase grows or melts. We conclude that the dynamics of the system becomes extremely lower when the initial amount of NaCl in the aqueous phase is increased. This is provoked by two effects. The first one is the concentration of NaCl in the aqueous phase, which probably decreases the water disponibility due to a solvation effect. The second one is because simulation temperatures become lower due to the cryoscopic decrease effect. As a result of both effects, simulation times required in the absence of NaCl are ≈10–20 shorter than those required at NaCl 3.0 m. As a consequence, the simulations of this work added together, result in a total simulation time of about 300 μs.

In summary, this work provides an accurate description of the *T*_3_ and the cryoscopic decrease effect determination of the CO_2_ hydrate at different pressures. We validate the use of the Madrid-2019 force fields (salt + TIP4P/2005 water models) for the study of the cryoscopic decrease effect in the particular case of hydrates. This finding contributes to the understanding of the stability conditions of hydrates under realistic seawater conditions and, together with the previous results obtained by some of the authors,^29^ leads the way to explore other hydrates using the Madrid-2019 force field.

## Data Availability

The data that support the findings of this study are available within the article.
